# The Ensembl REST API: Ensembl Data for Any Language

**DOI:** 10.1093/bioinformatics/btu613

**Published:** 2014-09-17

**Authors:** Andrew Yates, Kathryn Beal, Stephen Keenan, William McLaren, Miguel Pignatelli, Graham R. S. Ritchie, Magali Ruffier, Kieron Taylor, Alessandro Vullo, Paul Flicek

**Affiliations:** ^1^European Molecular Biology Laboratory, European Bioinformatics Institute, Wellcome Trust Genome Campus, Hinxton, Cambridge CB10 1SD and ^2^Wellcome Trust Sanger Institute, Wellcome Trust Genome Campus, Hinxton, Cambridge CB10 1SA, UK

## Abstract

**Motivation:** We present a Web service to access Ensembl data using Representational State Transfer (REST). The Ensembl REST server enables the easy retrieval of a wide range of Ensembl data by most programming languages, using standard formats such as JSON and FASTA while minimizing client work. We also introduce bindings to the popular Ensembl Variant Effect Predictor tool permitting large-scale programmatic variant analysis independent of any specific programming language.

**Availability and implementation:** The Ensembl REST API can be accessed at http://rest.ensembl.org and source code is freely available under an Apache 2.0 license from http://github.com/Ensembl/ensembl-rest.

**Contact:** ayates@ebi.ac.uk or flicek@ebi.ac.uk

**Supplementary information:** Supplementary data are available at *Bioinformatics* online.

## 1 INTRODUCTION

Ensembl data (Flicek *et al.*, 2014) are accessible in a variety of ways including our genome browser, BioMart data-mining tool (Kinsella *et al.*, 2011), the Bioconductor R package (Gentleman *et al.*, 2004) or viewers such as Dalliance (Down *et al.*, 2011). Direct programmatic access has historically required a native client interacting with the database in its own programming language. This solution required the reimplementation of functionality across multiple languages, which was costly to maintain and partly led to our focus only on a Perl API for Ensembl. Third-party Ensembl API bindings do exist, but may struggle to keep pace with new developments resulting in possible out-of-date implementations.

Remote procedure calling and Web services are a widely accepted solution to provide a single programming interface to multiple languages. SOAP is one such popular technology (McWilliam *et al.*, 2013) but is burdened with significant setup and processing overhead for the client. Newer Web services are based on the Representational State Transfer (REST) pattern (Fielding, 2000). REST encourages the reuse of HTTP technology to send and receive data in the same way a Web browser requests and receives a Web page via uniform resource locators (URLs). REST imposes no format restrictions on the returned data. The Distributed Annotation System (Jenkinson *et al.*, 2008) is an attempt to design a generic REST system for a biological 'data, but necessitated custom client libraries coupled with an XML format seen as verbose and inflexible.

We present a set of REST bindings to access Ensembl data and tools, exposing these data in simple formats that are well understood by a large proportion of programming languages.

## 2 IMPLEMENTATION

### 2.1 The REST API

Ensembl REST API calls are based on simple URLs that specify both the data required and the format returned. For example, to request the protein sequence for BRCA2 (ENSP00000439902) as a JSON document, it is only necessary to enter the following URL in a Web browser: http://rest.ensembl.org/sequence/id/ENSP00000439902.json

The components of the URL define the desired data and/or action. For example, /sequence performs sequence retrieval while /vep provides access to the results of the Ensembl Variant Effect Predictor (VEP) (McLaren *et al.*, 2010). With similar commands, users can retrieve features such as genes, orthologs, variants, genomic alignments and gene trees or perform actions such as convert coordinates between assemblies among other actions.

Incorporating Ensembl data into any analysis requires a HTTP library and a JSON parser. HTTPS is supported for secure client access. Below is an example of a request from Python to print number of variants that overlap with BRAF (ENSG00000157764):

import requests
url =
‘http://rest.ensembl.org/overlap/id/ENSG00000157764.json?feature=variation’
r = requests.get(url)
if not r.status_code == 200:
    raise Exception(‘Bad response’)
print len(r.json())

Required parameters are embedded within the URL. In our first example, ENSP00000439902 was a required parameter. Optional parameters are specified as simple ‘*key = value*’, pairs appended to a URL. Whenever possible, the server infers parameters from others. For example, species is determined from an Ensembl gene or a transcript stable ID. HTTP headers can be used to control the output format or enable on-the-fly gzip compression. Each endpoint emits four output formats: JSON, XML, YAML and JSON-P in addition to formats such as PhyloXML (Han and Zmasek, 2009).

Clients are rate limited on our public REST server to 15 requests per second, i.e. 54 000 requests per hour. To enable users to manage this or any future limits, each call to the REST server returns a number of HTTP response headers describing the IP address’s current limits and how long before this limit resets. Once exceeded, a Retry-After header is sent back, and the client is expected to sleep for this period before making a new request.

All endpoints are accompanied by automatically generated documentation available from http://rest.ensembl.org. Each page details parameters with brief descriptions and example values. Example URLs are shown alongside their output and example clients. These clients are written in popular programming languages, such as Python, Perl and Ruby. A higher-level user guide detailing version migration guides, best practices and more advanced clients is also provided at https://github.com/Ensembl/ensembl-rest/wiki.

### 2.2 Large-scale variant annotation

During our beta phase for the REST API, we observed significant traffic coming from requests to the VEP endpoint and annotating human variation. In fact, we noticed single IP addresses sending ∼3 million requests to the VEP endpoint, which we interpreted as full annotation of human variomes. This process is inefficient: using HTTP GET and rate limited to 15 requests per second, it would take 2.3 days annotate a human variome.

In response to this use case, we extended the Ensembl REST API to allow the submission of up to 1000 variants in a single HTTP POST. Adoption of VEP’s offline cache files enables our service to annotate large quantities of variants without exceeding HTTP time-outs. Benchmarking (Table 1) has shown annotation rates of ∼1000 variants per second for a single sample (HG00096) extracted from 1000 Genomes Phase 1 data (The 1000 Genomes Project Consortium, 2012). Analysis of a human variome within an hour is feasible using our public server.

**Table 1.** Benchmarking 3.5 million non-synonymous single nucleotide variants from three Amazon Elastic Compute Cloud (EC2) locations

EC2 location	Elapsed time (s)	Variants per second
Ireland	3631	946.66
VA, USA	3508	998.59
Singapore	5385	652.45

Benchmarks are averaged over three runs with a single Perl program with nine concurrent connections.

## 3 DISCUSSION

The Ensembl REST API can be used to query the Ensembl data resources and tools from a variety of programming languages and enables flexible programmatic access previously only supported by our Perl API. The reduced setup costs for a client means that users can interact with the latest Ensembl data without the need to follow our regular API releases. Supporting POST requests for VEP enables the annotation of large-scale variation datasets without the need to download or host the VEP code or cache files. HTTP has also proven a more robust data protocol when compared with MySQL improving user experience for worldwide users.

A number of native third-party APIs have been developed to help access the REST API in languages such as Python, R and JavaScript, which demonstrates the usefulness of our REST API to these increasingly popular bioinformatics languages. JavaScript applications such as Wasabi (http://wasabiapp.org) import Ensembl Gene Trees and genome multiple sequence alignments via REST creating a seamless link between tool and data. RNACentral (Bateman *et al.*, 2011) displays non-coding gene models alongside Ensembl annotation in Genoverse (http://genoverse.org/), a HTML5 genome browser, using data from our REST API.

REST has shown itself to be a sustainable model for the distribution of genomic data to multiple programming languages. We plan to expand the coverage of Ensembl data and tools hosted in it. We also plan to provide more formats from the service such as VCF output from our VEP endpoint. We will continue to work with tool developers to ensure the service is suitable for their purposes.

## Supplementary Material

Supplementary Data
