# Supplementary material for: The Ensembl REST API: Ensembl Data for Any Language
Source: Bioinformatics. 2014 Sep 17;31(1):143–5. doi: 10.1093/bioinformatics/btu613 (PMC4271150; doi:10.1093/bioinformatics/btu613)
Supplement: Supplementary Data [file supp_31_1_143__index.html]

The Ensembl REST API: Ensembl Data for Any Language — The Ensembl REST API: Ensembl Data for Any Language — The Ensembl REST API: Ensembl Data for Any Language — Supplementary Data 

# The Ensembl REST API: Ensembl Data for Any Language

## Supplementary Data

files

**Files in this Data Supplement:**

- Supplementary Data - doc file
