# Supplementary material for: The Ensembl REST API: Ensembl Data for Any Language
Source: Bioinformatics. 2014 Sep 17;31(1):143–5. doi: 10.1093/bioinformatics/btu613 (PMC4271150; doi:10.1093/bioinformatics/btu613)
Supplement: Supplementary Data [file supp_btu613_README.doc]

# Supplemental Material - The Ensembl REST API: Ensembl Data for Any Language

## Benchmarking of the REST API for variation annotation

### Dependencies

The code requires Perl 15.4.2 (or upwards) and a number of 3rd party Perl library dependencies. These have been specified in the accompanying cpanfile. To install with cpanminus <http://search.cpan.org/dist/App-cpanminus/bin/cpanm> use the following command.

> cpanm --installdeps .

You also need a copy of VCFTools (<http://vcftools.sourceforge.net/>) and Tabix (<http://samtools.sourceforge.net/tabix.shtml>) to perform the data slicing in step 2. Please follow instructions from their respective sites to install the binaries.

### Step 1: Downloading 1000 Genomes Data

Data was taken from 1000 Genomes phase 1 data from <http://ftp.1000genomes.ebi.ac.uk/vol1/ftp/release/20110521>. These files were transferred to a local disk using Globus using the instructions at <http://www.1000genomes.org/category/frequently-asked-questions/globus>. You should download all VCF files in this directory.

### Step 2: Extracting nonsynonymous SNVs for sample HG000096

The script **extract_sample.sh** processes any file with the pattern **ALL.chr*.snps_indels_svs.genotypes.vcf.gz** passing the data through vcftools. Each processing job was submitted to our local compute farm via LSF and used a high performance IO system to read VCF from and write it back to (memory requirements are minimal). Please run **extract_sample.sh** to run the same analysis and edit it to your personal requirements.

### Step 3: Benchmarking

Benchmarking was run from multiple Amazon Web Services locations using a m3.xlarge server (4 vCPUs, 15 GB RAM and 80GB SSD storage). We chose the following (no sub-zone selection was specified):

- Virginia, USA

- Ireland

- Singapore

**rest-benchmark.sh** coordinates the benchmarking run. The following example runs 9 benchmarks from 1 concurrent clients to 9:

> gunzip HG00096.vcf.gz

> ./rest-benchmark.sh embassy HG00096.vcf 9

Batch size is set to 1000 lines of VCF and must run on a decompressed VCF file. Each run will produce a summary of data to screen which is generated by the **stats.pl** script. Also the results of benchmarking are held in a file called **logs${concurrent_clients}.db** e.g. **logs9.db**. The number refers to the number of concurrent clients ran. **view_log.pl** can be used to view the data in each individual log structure if required.

All code was rerun 3 times in each zone to produce the final published averages.
